# Mifepristone Access Through Community Pharmacies When Regulated as a Routine Prescription Medication

**DOI:** 10.1001/jamanetworkopen.2025.42096

**Published:** 2025-11-06

**Authors:** Elizabeth Nethery, Catherine Xu, Carissa S. Y. Chan, Mary Helmer-Smith, Andrea Stucchi, Dawn Mooney, Enav Z. Zusman, Sheila Dunn, Robert Pammett, Wendy V. Norman, Giuliana Guarna, Michael R. Law, Laura Schummers

**Affiliations:** 1 Collaboration for Outcomes Research and Evaluation, Faculty of Pharmaceutical Sciences, The University of British Columbia, Vancouver, British Columbia, Canada; 2 Faculty of Medicine, The University of British Columbia, Vancouver, British Columbia, Canada; 3 Centre for Health Services and Policy Research, The University of British Columbia, Vancouver, British Columbia, Canada; 4 Department of Obstetrics & Gynaecology, Faculty of Medicine, University of British Columbia, Vancouver, British Columbia, Canada; 5 Department of Family & Community Medicine, University of Toronto, Toronto, Ontario, Canada; 6 Women’s College Research Institute, Women’s College Hospital, Toronto, Ontario, Canada; 7 Faculty of Pharmaceutical Sciences, The University of British Columbia, Vancouver, British Columbia, Canada; 8 Northern Health Authority, Prince George, British Columbia, Canada; 9 Department of Family Practice, Faculty of Medicine, The University of British Columbia, Vancouver, British Columbia, Canada; 10 Department of Public Health, Environments and Society, Faculty of Public Health and Policy, London School of Hygiene & Tropical Medicine, London, United Kingdom; 11 Department of Obstetrics & Gynaecology, McMaster University, Hamilton, Ontario, Canada; 12 Department of Community Health Sciences, University of Calgary, Calgary, Alberta, Canada; 13 Centre for Health Policy, O’Brien Institute for Public Health, University of Calgary, Alberta, Canada

## Abstract

**Importance:**

The mifepristone-misoprostol medication abortion regimen became available in Canada as a normally prescribed medication, without restrictions, in 2017. Despite rapid uptake, patients have reported difficulty accessing this time-sensitive medication.

**Objective:**

To quantify mifepristone availability within 3 calendar days at community pharmacies.

**Design, Setting, and Participants:**

This population-based, cross-sectional study using a mystery caller survey was conducted from July to August 2024 at community pharmacies in British Columbia (BC), Canada.

**Exposure:**

Surveyor posing as a patient with a prescription for mifepristone who requests to pick up the medication within 3 calendar days. For nondispensing pharmacies (ie, unable to dispense within 3 days), surveyors requested a referral to a dispensing pharmacy.

**Main Outcomes and Measures:**

Proportion of pharmacies that could dispense mifepristone or provide a valid referral to a dispensing pharmacy; proportion of reproductive-aged (15-49 years) female population residing within 15-minute walking and 15-, 30-, and 60-minute driving times of a dispensing pharmacy, and proportion of the population with poor local availability (<50% of available pharmacies dispense mifepristone).

**Results:**

Among 1460 of 1482 community pharmacies, 962 (66%) could dispense mifepristone within 3 days and 169 (12%) provided a valid referral. Only one-third of nondispensing pharmacies (169 of 498 pharmacies [34%]) provided a valid referral to a dispensing pharmacy; 329 pharmacies (23%) neither dispensed within 3 days nor provided a valid referral. Almost all reproductive-aged females (1 095 915 of 1 110 218 females [99%]) in BC lived within a 15-minute drive of a mifepristone-dispensing pharmacy. Urban pharmacies were more likely to be nondispensing and nonreferring (relative risk [RR], 1.78; 95% CI, 1.19-2.80) and less likely to offer same-day dispensing (RR, 0.39; 95% CI, 0.30-0.51) compared with rural pharmacies. Populations in areas with the most residential instability (RR, 1.41; 95% CI, 1.19-1.67) or greater ethnocultural diversity (RR, 1.36; 95% CI, 1.15-1.61) had poorer local availability of mifepristone-dispensing pharmacies.

**Conclusions and Relevance:**

This cross-sectional study found 99% of reproductive-aged BC females lived within a 15-minute drive of a mifepristone-dispensing pharmacy; however, despite relatively good geographic coverage and mifepristone being available as a routine prescription, 23% of pharmacies neither dispensed within 3 days nor provided a valid referral, putting the onus on patients to identify a dispensing pharmacy. These findings may inform policy and initiatives to enhance pharmacist referral networks and improve mifepristone access, as well as service planning for international jurisdictions considering a similar medication abortion framework.

## Key Points

**Question:**

What proportion of community pharmacies dispense mifepristone within 3 calendar days when mifepristone is regulated as a routine prescription medication?

**Findings:**

This cross-sectional study using a mystery caller survey of 1460 pharmacies in British Columbia, Canada, found that 66% of pharmacies could dispense mifepristone within 3 days and 12% referred to another pharmacy that could.

**Meaning:**

These findings suggest that when medication abortion is available as a routine health service and mifepristone is regulated as a routine prescription, pharmacists play a key role in providing geographically distributed access to medication abortion; access could be improved through policies and initiatives that enhance and support pharmacist referral networks.

## Teaser

This cross-sectional study quantifies the availability of mifepristone within 3 calendar days at community pharmacies across British Columbia, Canada.

## Introduction

Canada decriminalized abortion in 1988 and has established abortion care as a routine health service.^1^ Abortion provision in Canada, including medication abortion, is regulated the same as any health service or medical procedure. Thus, abortion care is not specified in criminal law but is managed by general provincial legislation and regulations governing authorization and licensing for health care professionals, and accreditation for facilities. In 2017, mifepristone was introduced in Canada. Prior to this, medication abortion was provided using methotrexate and misoprostol or misoprostol alone,^2,3^ and more than 96% of abortions were procedural.^4^ The introduction of mifepristone^5,6^ in Canada and the rapid removal of regulatory barriers, such as observed dosing (ie, the physician must observe the patient swallowing the mifepristone) and physician-only dispensing,^7^ increased uptake of medication abortion from slightly more than 3% of all abortions in 2016^4^ to 40% by 2022.^8,9^ In Canada’s current model, pharmacists dispense mifepristone directly to patients who present with a standard prescription. When Canada introduced this unprecedented medication abortion model, abortion remained safe with persistently low risks of complications or adverse events.^8,10^ Routine pharmacist dispensing of mifepristone may facilitate timely access to abortion care, reducing risks of complications^11^ that rise with increasing gestational age.^12^

While provision of medication abortion has increased rapidly,^7,13,14^ patients and prescribers have reported difficulty finding a pharmacy that stocks and/or dispenses this medication.^15,16,17,18,19^ Reasons for not stocking mifepristone include high inventory carrying costs, short expiry date, refusal to carry the medication, and infrequent demand.^15,18,20^ Although Canada’s single-payer provincial health insurance programs universally cover the medication costs^1^ (since 2019), pharmacies are reimbursed only after dispensation.^21^ Thus, some pharmacies report not regularly stocking mifepristone, instead purchasing from another pharmacy or ordering as needed.^18^ A recent population-based study in Ontario, Canada found that 20% of all pharmacies filled at least 1 mifepristone prescription in 2022 (increased from 2% in 2017).^22^ However, this study lacked data on the frequency of mifepristone requests and thus could not disentangle pharmacy ability to dispense from mifepristone demand. By contrast, when callers posed as physicians or medical students asking on behalf of a patient, between 6% to 13% of pharmacies reported current stock of mifepristone.^15,20^

Our study focused on the patient-level experience of obtaining mifepristone following a routine prescription, given a reasonable time frame of pharmacy prescription fulfillment. To comprehensively evaluate access to mifepristone at community pharmacies, the objectives of this study were to determine the number and geographic distribution of pharmacies in British Columbia (BC) that can dispense mifepristone within 3 days and the validity of referrals from nondispensing pharmacies. We also sought to identify population-level gaps in access to this time-sensitive medication.

## Methods

This cross-sectional study received research ethics approval from the University of British Columbia Behavioral Research Ethics Board. Reporting follows the Strengthening the Reporting of Observational Studies in Epidemiology (STROBE) reporting guideline.

### Telephone Survey

We conducted a cross-sectional telephone survey of all pharmacies in BC from July to August 2024 using a mystery caller approach.^15,23^ A mystery caller (or secret shopper) design uses simulated clients to assess service delivery and is a method increasingly used in health care service delivery research.^24,25,26^ We identified all BC pharmacies using the Licensed Community Pharmacy and Licensed Telepharmacy Directories on the College of Pharmacists of British Columbia public website^27^ and extracted pharmacy names, locations (including postal codes and street addresses), and telephone numbers.

Two surveyors (C.C. and C.X.) posed as patients seeking to fill a prescription for mifepristone. Pharmacy calls were made during any day of the week (July 1 to August 31, 2024). The survey was conducted with the first pharmacy staff member who picked up the telephone or to whom the surveyor was transferred. Both surveyors followed a predetermined script (eMethods in Supplement 1) and entered the responses in real time into a form on the secure Qualtrics survey platform. If the pharmacy could fill the prescription, surveyors asked whether it could be dispensed within 3 days. Our study team pharmacists defined 3 days as the maximum period for timely dispensing because this balanced the time-sensitive patient need for mifepristone for medication abortion (or off-label miscarriage management^28^) and allowed reasonable time for fulfilling an order if the medication was not in stock (including weekends and holidays), based on study pharmacists’ knowledge of supply chain and delivery timelines in BC. If the pharmacy was unable to fill the prescription within 3 days, the surveyor requested a referral to another pharmacy that could fill the prescription. Data were not obtained regarding dispensing time when a pharmacy was unable to dispense within 3 days nor reasons for nondispensing or delayed dispensing. No identifying information was requested from the responding pharmacy employee. Any identifying information disclosed by the respondent was not recorded. The use of deception and exemption from the requirement to debrief participants in this study were carefully considered and met the conditions outlined in Canadian research ethics guidance.^29^

### Other Data Sources

We classified pharmacies as corporate (pharmacy within a large corporate business [ie, grocery store chain or large retail chain]), banner or franchise (pharmacy owned or operated by a franchise owner and/or using a licensed brand), or independent (independently owned with no corporate, franchise, or banner involvement) using publicly available information. We geocoded^30^ pharmacy locations using their registered street address and mapped locations to BC health regions and community health service areas (CHSAs).^31^ CHSAs are community level geographic areas nested within larger health regions in BC and are classified (urban to remote) to represent access to health services. We linked census dissemination area (DA) boundaries with population estimates of reproductive-aged females (ages 15 to 49 years) based on the 2021 Canadian census^32^ and to indicators of socioeconomic deprivation and marginalization from the Canadian Index of Multiple Deprivation (CIMD).^33^ DAs are the smallest Canadian geographic unit for which sex- and age-stratified population estimates are publicly available and represent approximately 400 to 700 people. We spatially intersected DAs with CHSAs to obtain rural-urban DA classifications. When DAs overlapped more than 1 CHSA, we assigned urban-rural classifications based on the area with the largest portion of the DA.

The CIMD measures 4 key dimensions of deprivation and marginalization for each DA. These scores are calculated using principal component analysis from a pool of 32 input census variables. Economic dependency relates to workforce participation, or a dependence on sources of income other than employment income; residential instability speaks to the tendency of neighborhood inhabitants to fluctuate over time, taking into consideration both housing and familial characteristics; ethnocultural composition refers to the community make-up of immigrant populations; and situational vulnerability speaks to variations in sociodemographic conditions in the areas of housing and education, while taking into account other demographic characteristics. While the CIMD represents area-level inequity and marginalization, it can also be a proxy for individual-level inequity and deprivation.^33^ Each score is reported as quintiles from lowest (least inequity or least diversity) to highest (most deprived, most diverse, or highest inequity).

### Outcome Variables and Geographic Analyses

First, we classified pharmacies as (1) mifepristone-dispensing pharmacies (pharmacies that could dispense within 3 days), (2) nondispensing referring pharmacies (pharmacies that could not dispense either within 3 days or at all but provided a valid referral to a dispensing pharmacy), and (3) nondispensing, nonreferring pharmacies (pharmacies that could not dispense either within 3 days or at all and did not provide a valid referral). Referrals were validated by cross-checking information provided by the respondent to identify the referral pharmacy (name, address, and telephone number) and using our survey results to confirm the dispensing status of the referral pharmacy. We defined invalid referrals as a referral to a nonexistent or unidentifiable pharmacy or to a nondispensing pharmacy.

To combine our pharmacy-level data with the geographic distribution of populations in our province, we used network analysis to generate service areas based on travel times from all geocoded pharmacy locations using road networks. In ArcGIS Pro,^34^ we constructed service areas based on 15-minute walk times, and 15-minute, 30-minute, and 60-minute driving times for each pharmacy; intersected these service areas with DAs; and counted the total number of mifepristone-dispensing pharmacies and all pharmacies (dispensing or nondispensing) for each DA.^35^ Next, we defined 2 outcomes: a variable representing the closest travel time to a mifepristone-dispensing pharmacy (15-minute walk and 15-minute drive, 30-minute drive, or 60-minute drive) and a variable representing the local availability of mifepristone-dispensing pharmacies within a reasonable travel time (poor vs good availability).

For the outcome of local availability, travel time service areas were assigned according to the population density of each DA. Longer travel times were used for more sparsely populated rural or remote areas and shorter travel times for highly populated urban areas.^36^ Specifically, for high density DAs (defined as >1000 persons per km^2^), we used service areas with 15-minute walk times; for medium density DAs (100 to 999 persons per km^2^), we used 15-minute drive times; and for low density areas (<100 people per km^2^), we used 30-minute drive times. If no pharmacies existed within the density-specified travel time, we used the next travel time level. For example, high density urban areas without a pharmacy within a 15-minute walk would be assessed using a 15-minute drive time. We then calculated the proportion of mifepristone-dispensing pharmacies relative to the total number of pharmacies within the most reasonable travel time area for that DA. This yielded a single outcome variable representing the local availability of a mifepristone-dispensing pharmacy valid for each DA, with poor availability defined as less than 50% of pharmacies within a reasonable travel time. The results (closest travel time and local availability) were weighted by the population of reproductive aged females in each DA, yielding population estimates for both outcomes.

### Statistical Analysis

We calculated proportions with 95% CIs of mifepristone availability (dispensation possible within 3 calendar days, nondispensing with valid referral, nondispensing, and no valid referral) at all pharmacies, and stratified results by health region, pharmacy type, and rural-urban status. We estimated proportions of reproductive-aged females residing in DAs for both DA level outcomes: closest mifepristone-dispensing pharmacy and poor local availability (<50% of pharmacies dispense). Results were stratified by DA subgroups (rural-urban and region). For local availability, we also stratified by area-level deprivation indicators. We used unadjusted, bivariable regression models (binomial Poisson) to estimate relative risks (RRs) for mifepristone-dispensing (pharmacy level) and population proportions (DA level) for subgroups compared with baseline groups with 95% CIs and generated maps for visualization. Statistical analysis and maps were completed in R version 4.50 (R Project for Statistical Computing).^37^ In secondary analyses, we calculated the proportion of dispensing pharmacies that could dispense today or today/tomorrow, referral validity among nondispensing pharmacies, and the mean proportion of available pharmacies for each DA and travel time.

## Results

We identified 1511 pharmacies from the College of Pharmacists of BC publicly available list of community pharmacies. We excluded 29 atypical pharmacies that reported only dispensing to other pharmacies (eg, central fill, bulk medication compounding, and fulfillment to long-term care facilities) or specific medication classes (eg, biologic medications or those used to treat substance use disorders). Of the remaining 1482 pharmacies meeting our inclusion criteria, we successfully contacted and surveyed 1460 pharmacies (99% response rate) (eFigure 1 in Supplement 1). Of all surveyed pharmacies, 1131 of 1460 (78%) could dispense mifepristone within 3 calendar days (962 pharmacies [66%]) or referred to a valid dispensing pharmacy (169 pharmacies [12%]), and 329 (23%) were unable to dispense within 3 days and did not provide a valid referral. Of the 962 pharmacies that could dispense within 3 days, 806 (84%) could dispense on the same or next day. Pharmacies in urban areas were more likely to be nondispensing and nonreferring pharmacies compared with pharmacies in rural or remote areas (RR, 1.78; 95% CI, 1.19-2.80; *P* = .008) (Table 1).

**Table 1.** Mifepristone Dispensing, Referral, and Nondispensing at Community Pharmacies in British Columbia, Canada (N = 1460)

Characteristic	Pharmacy can dispense mifepristone in 3 d (timely dispensing; n = 962)			Pharmacy is nondispensing but gave valid referral (n = 169)			Pharmacy is nondispensing and nonreferring (n = 329)		
	% (95% CI)^a^	RR (95% CI)	*P* value	% (95% CI)^a^	RR (95% CI)	*P* value	% (95% CI)^a^	RR (95% CI)	*P* value
Overall	66 (63-68)	NA	NA	12 (10-13)	NA	NA	23 (20-25)	NA	NA
Pharmacy type^b^									
Corporate or chain	66 (61-70)	1 [Reference]	NA	11 (8-14)	1 [Reference]	NA	23 (19-28)	1 [Reference]	NA
Banner or franchise	68 (64-71)	1.04 (0.89-1.20)	.65	11 (9-14)	1.03 (0.72-1.49)	.88	21 (18-24)	0.89 (0.69-1.15)	.36
Independent	61 (56-67)	0.93 (0.78-1.12)	.47	13 (10-17)	1.20 (0.78-1.82)	.41	26 (21-31)	1.09 (0.81-1.47)	.55
CHSA rural-urban type^c^									
Rural hub, rural, or remote	77 (69-83)	1 [Reference]	NA	9.1 (5-15)	1 [Reference]	NA	14 (9-21)	1 [Reference]	NA
Medium or small urban	67 (61-72)	0.87 (0.70-1.08)	.21	14 (10-18)	1.51 (0.86-2.81)	.17	19 (15-24)	1.39 (0.87-2.29)	.18
Metropolitan or large urban	64 (61-67)	0.83 (0.69-1.01)	.057	11 (9-13)	1.23 (0.74-2.20)	.45	25 (22-28)	1.78 (1.19-2.80)	.008
Region^d^									
Metropolitan Vancouver	65 (62-68)	1 [Reference]	NA	11 (9-13)	1 [Reference]	NA	24 (21-27)	1 [Reference]	NA
Metropolitan Victoria	57 (47-66)	0.87 (0.66-1.12)	.30	13 (8-22)	1.24 (0.68-2.10)	.45	30 (22-40)	1.24 (0.84-1.78)	.25
Medium cities	67 (61-73)	1.04 (0.87-1.22)	.68	14 (10-19)	1.33 (0.90-1.93)	.14	18 (14-24)	0.76 (0.55-1.03)	.08
Other areas	72 (65-79)	1.11 (0.91-1.35)	.29	11 (7-16)	1.00 (0.58-1.61)	.99	17 (12-24)	0.70 (0.47-1.02)	.08
Northern areas	69 (55-80)	1.06 (0.76-1.44)	.72	14 (7-26)	1.30 (0.58-2.50)	.48	17 (9-30)	0.71 (0.35-1.27)	.29

Abbreviations: CHSA, community health service area; NA, not applicable; RR, relative risk.

^a^ Row % (95% CI).

^b^ Pharmacies classified as corporate (pharmacy within a large corporate business [ie, grocery store chain or large retail chain]), banner, or franchise (pharmacy owned or operated by a franchise owner and/or using a licensed brand), or independent (independently owned with no corporate interests, franchise, or banner) using publicly available information.

^c^ CHSAs were obtained by mapping pharmacy locations onto CHSA boundaries; rural-urban classifications for each CHSA are obtained from the provincial health agencies.

^d^ Regions are broadly defined based on regional health authority areas. Metropolitan Vancouver includes all local health areas within the greater Vancouver metropolitan area. Metropolitan Victoria includes all local health areas in the greater Victoria area. Medium cities includes all local health areas that include cities with regional hospitals. Northern areas includes all areas outside of Prince George in Northern British Columbia. Other areas includes all other areas outside the medium cities or large metropolitan areas.

Among all 498 nondispensing pharmacies, 169 (34%) provided a valid referral, and we observed no differences in valid referrals by pharmacy type or rural-urban status (eTable 1 in Supplement 1). Among the 329 nondispensing and nonreferring pharmacies, 3 (1%) referred to a sexual health clinic, 59 (18%) provided a specific referral to another nondispensing pharmacy, 58 (18%) gave a vague referral (eg, call around or try a chain or named pharmacy), and the majority (209 pharmacies [64%]), provided no referral (eTable 2 in Supplement 1). Pharmacies in the metropolitan or large urban areas were least likely to be able to dispense mifepristone on the same day (RR, 0.39; 95% CI, 0.30-0.51; *P* < .001) (eTable 3 in Supplement 1).

At the DA-level, on average, 63% (95% CI, 63%-64%) of pharmacies within a 15-minute drive of an urban DA dispensed mifepristone compared with 74% (95% CI, 72%-75%) of pharmacies dispensing within a 15-minute drive of rural DAs (RR, 0.86; 95% CI 0.85-0.87; *P* < .001) (eTable 4 in Supplement 1). Three percent of DAs in BC (232 of 7848 DAs) had no pharmacy within a 60-minute drive, representing less than 1% of the reproductive-aged female population (3453 of 1 113 671 females) (eTable 5 in Supplement 1). Among all reproductive-aged females residing in DAs with a pharmacy within a 60-minute drive, most (869 427 of 1 110 218 females [94%]) lived within a 15-minute walk of at least 1 mifepristone-dispensing pharmacy, and the vast majority (1 095 915 of 1 110 218 females [99%]) lived within at least a 15-minute drive (Table 2 and eFigure 2 in Supplement 1). While most urban females (692 306 of 801 283 females [86%]) had a dispensing pharmacy within a 15-minute walk, less than one-half (53 103 of 122 841 [43%]) had similar access in rural areas. All 801 283 urban females (100%) lived within a 15-minute drive of a dispensing pharmacy compared with rural females (109 967 of 122 841 females [90%]). A small population (7496 of 122 841 females [6%]) in rural areas had no pharmacy within a 15-minute drive.

**Table 2.** Estimated Population of Reproductive-Aged Females in BC, Canada By Travel Time to a Mifepristone-Dispensing Pharmacy

Characteristic	Closest mifepristone-dispensing pharmacy, No. (%)				No mifepristone-dispensing pharmacy within a 60-min drive, No. (%)	Total population of reproductive-aged females with at least 1 pharmacy within a 60-min drive, No. (%)
	15-min Walk	15-min Drive	30-min Drive	60-min Drive		
Overall	869 427/1 110 218 (78)	226 488/1 110 218 (20)	9688/1 110 218 (0.9)	3835/1 110 218 (<1)	780/1 110 218 (<1)	1 110 218
CHSA rural-urban type^a^						
Rural hub, rural, or remote	53 103 (43)	56 864 (46)	8473 (7)	3621 (3)	780 (1)	122 841 (11)
Medium or small urban	124 018 (67)	60 647 (33)	1215 (1)	214 (<1)	<5 (<1)	186 094 (17)
Metropolitan or large urban	692 306 (86)	108 977 (14)	<5 (<1)	<5 (<1)	<5 (<1)	801 283 (72)
Region^b^						
Metropolitan Vancouver	615 851 (87)	93 086 (13)	965 (<1)	57 (<1)	<5 (<1)	709 959 (64)
Metropolitan Victoria	64 850 (75)	21 741 (25)	315 (<1)	<5 (<1)	10 (<1)	86 916 (8)
Medium cities	118 656 (67)	57 604 (32)	1190 (1)	460 (<1)	105 (<1)	178 015 (16)
Other areas	52 460 (54)	37 241 (38)	5556 (6)	1872 (2)	664 (1)	97 793 (9)
Northern areas	17 610 (47)	16 816 (45)	1662 (4)	1446 (4)	<5 (<1)	37 535 (3)

Abbreviation: CHSA, community health service area.

^a^ CHSA areas were obtained by mapping pharmacy locations onto CHSA boundaries. Rural-urban classifications for each CHSA are obtained from the provincial health agencies.

^b^ Regions are broadly defined based on regional health authority areas. Metropolitan Vancouver includes all local health areas within the greater Vancouver metropolitan area. Metropolitan Victoria includes all local health areas in the greater Victoria area. Medium cities includes all local health areas that include cities with regional hospitals. Northern areas includes all areas outside of Prince George in Northern British Columbia. Other areas includes all other areas outside the medium cities or large metropolitan areas.

With the province’s population highly concentrated in large urban centers, most reproductive-aged females (924 193 of 1 113 671 females [83%]) lived in DAs with at least 1 pharmacy within a 15-minute walk. However, despite living within a 15-minute walk from a pharmacy, some (159 158 of 924 193 females [17%]) had poor local availability (<50% of available pharmacies) of mifepristone-dispensing pharmacies (eTable 6 in Supplement 1).

Reproductive-aged females in large metropolitan or urban areas had the highest risk of poor local availability (RR, 2.23; 95% CI, 1.73-2.88; *P* < .001) compared with rural or remote areas (Table 3). We found a higher risk of poor local availability in areas with the most residential instability (RR, 1.41; 95% CI, 1.19-1.67; *P* < .001) and areas with greater ethnocultural diversity (RR 1.36; 95% CI, 1.15-1.61; *P* < .001) compared with areas with the least instability or diversity (Table 3). Results were generally consistent using a single density-adjusted indicator and across individual travel times (Table 3 and eTable 6 in Supplement 1).

**Table 3.** Population of Reproductive-Aged Females in Dissemination Areas With Poor Local Availability^a^

Characteristic^b^	All reproductive-aged females, No.	Poor local availability of mifepristone-dispensing pharmacies^a^		
		No. (%)^c^	RR (95% CI)	*P* value
All	1 107 862	149 799 (14)	NA	NA
CHSA rural-urban type^d^				
Rural hub, rural, or remote	120 629	8048 (7)	1 [Reference]	NA
Medium or small urban	185 950	22 298 (12)	1.80 (1.35-2.39)	<.001
Metropolitan or large urban	801 283	119 453 (15)	2.23 (1.73-2.88)	<.001
Region^e^				
Metropolitan Vancouver	709 902	96 643 (14)	1 [Reference]	NA
Metropolitan Victoria	86 906	22 285 (26)	1.88 (1.56-2.28)	<.001
Medium cities	177 610	16 648 (9)	0.69 (0.54-0.88)	.002
Other areas	96 503	9595 (10)	0.73 (0.58-0.93)	.009
Northern areas	36 941	4628 (13)	0.92 (0.68-1.25)	.59
Canadian Index of Multiple Deprivation (socioeconomic indicators)^f^				
Economic dependency^g^				
Least dependency	566 290	73 350 (13)	1 [Reference]	NA
Most dependency	320 311	45 409 (14)	1.09 (0.94-1.28)	.26
Residential instability^h^				
Least instability	356 639	38 705 (11)	1 [Reference]	NA
Most instability	535 238	83 534 (15)	1.41 (1.19-1.67)	<.001
Ethnocultural composition^i^				
Least diversity	348 029	38 113 (11)	1 [Reference]	NA
Most diversity	542 905	81 010 (15)	1.36 (1.15-1.61)	<.001
Situational vulnerability^j^				
Least vulnerable	471 440	61 274 (13)	1 [Reference]	NA
Most vulnerable	400 432	56 730 (14)	1.09 (0.93-1.28)	.30

Abbreviations: CHSA, community health service area; NA, not applicable; RR, relative risk.

^a^ Less than 50% of all pharmacies in dissemination area were able to dispense mifepristone in 3 days.

^b^ Comparisons are for the most deprived 2 quintiles v the least deprived 2 quintiles with the middle quintile excluded.

^c^ No. (row %).

^d^ CHSA areas were obtained by mapping pharmacy locations onto CHSA boundaries, Rural-urban classifications for each CHSA are obtained from the provincial health agencies.

^e^ Regions are broadly defined based on regional health authority areas. Metropolitan Vancouver includes all local health areas within the greater Vancouver metropolitan area. Metropolitan Victoria includes all local health areas in the greater Victoria area. Medium cities includes all local health areas that include cities with regional hospitals. Northern areas includes all areas outside of Prince George in Northern British Columbia. Other includes all other areas outside the medium cities or large metro areas.

^f^ Missing data for local availability of mifepristone in Canadian Index of Multiple Deprivation subgroups for an estimated 365 individuals because of suppressed (low count) population data in these dissemination areas.

^g^ Economic dependency relates to reliance on the workforce or a dependence on sources of income other than employment income.

^h^ Residential instability speaks to the tendency of neighborhood inhabitants to fluctuate over time, taking into consideration both housing and familial characteristics.

^i^ Ethnocultural composition refers to the community make-up of immigrant populations.

^j^ Situational vulnerability speaks to variations in sociodemographic conditions in the areas of housing and education, while taking into account other demographic characteristics.

Geographic analyses further highlighted specific neighborhoods in large urban centers with very poor local availability (<25% of pharmacies) (Figure and eFigure 3 in Supplement 1) and corresponding to areas with greater ethnocultural diversity (Vancouver only), residential instability, and economic dependency (eFigure 4 in Supplement 1). Across the province, there were notable areas with a complete absence of pharmacies, resulting in poor local availability (Figure); however, the nearest mifepristone-dispensing pharmacy was accessible within a 30- or 60-minute drive (eFigure 2 in Supplement 1).

**Figure.**
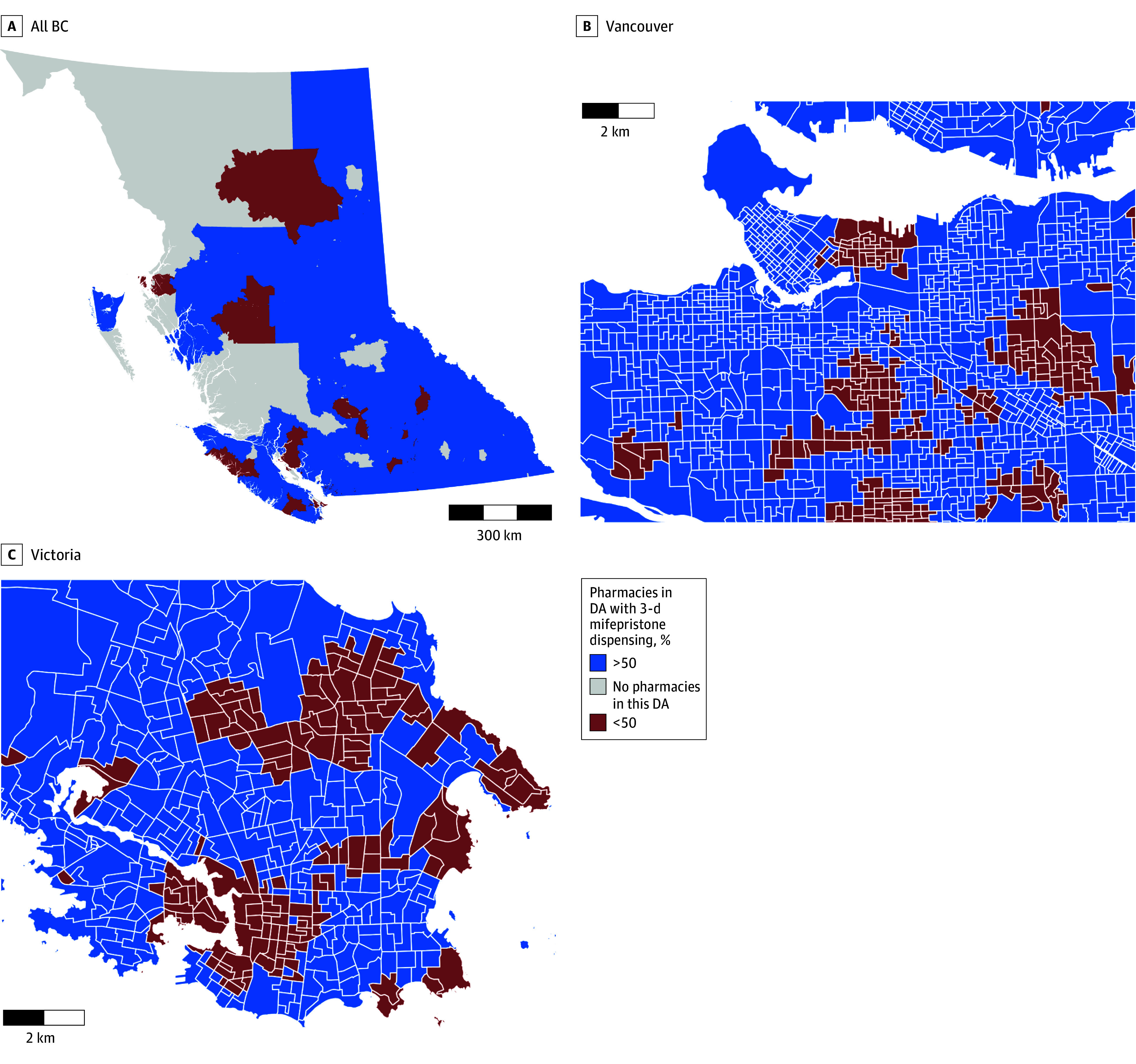
Maps of Dissemination Areas (DA) by Local Availability of 3-Day Mifepristone Dispensing Pharmacies as a Percentage of Available Pharmacies in Each DA BC indicates British Columbia.

## Discussion

In this population-based cross-sectional study using a mystery caller survey, more than three-quarters of all community pharmacies in BC, Canada could dispense mifepristone within 3 calendar days of receiving a prescription or provided a valid referral to one that could. Thus, nearly all (99%) reproductive-aged females in BC live within a 15-minute drive of at least 1 mifepristone-dispensing pharmacy. These findings suggest that, when mifepristone is regulated as a routine prescription medication (such that pharmacists can dispense without medication-specific restrictions), community pharmacies can serve a central role in facilitating local access to medication abortion across both urban and rural settings.

While finding a dispensing pharmacy was a barrier in the initial years of mifepristone availability in Canada,^7,16,17,38^ mifepristone was widely available through community pharmacies in BC within 7 years after regulatory change. Nevertheless, we identified several areas where coordination of services would further improve access. Of nondispensing pharmacies, only one-third referred the patient to a dispensing pharmacy. Not all pharmacies need to dispense mifepristone if referral networks between pharmacies are well-established and maintained. When referral pathways are unclear or inaccurate, patients must find a dispensing pharmacy on their own—highlighting the need for improved pharmacy referral networks. Our study aligns with patients’ experiences in dense urban areas of being advised to call around to find a dispensing pharmacy.^15^

The need for referral pathways was most clear in urban centers. Even with a high concentration of pharmacies in major urban centers (eg, Vancouver and Victoria), a lower proportion of pharmacies dispensed mifepristone. In these urban centers (together accounting for approximately 70% of the provincial population), we found poor local availability (<50% dispensed mifepristone) in several neighborhoods and in areas with greater ethnocultural diversity and socioeconomic inequity. Conversely, while there were fewer pharmacies in rural areas, more areas had at least 1 dispensing pharmacy and a greater proportion dispensed mifepristone. Rural pharmacists might have established working relationships with medication abortion prescribers or may keep a local stock of key medications due to longer medication acquisition times.^39,40^ In contrast, nondispensing status or poor referrals among urban pharmacies could be due to high volume practices (lack of time) or assumptions that nearby pharmacies have stock (eg, 18% of nondispensing pharmacies told the surveyor to call around without providing a specific referral).

The College of Pharmacists of BC criteria for referral emphasizes reasonableness with respect to referring a patient to another pharmacy^41^ and thus, while the referral patterns we observed may represent usual pharmacy practices (especially in urban areas), these do not provide optimal patient experiences.^42^ For patients already experiencing inequities, vulnerabilities (ie, youth, racially or ethnically minoritized, or immigrant status), or low health literacy, inadequate referrals could create additional barriers, delay access to abortion care, and exacerbate inequities.^43^

Recent studies have also shown an overall increase in use of medication (vs procedural) abortion in Canada,^22^ more uptake in primary care,^44,45^ and increasing rural provision.^13,14^ Medication abortion is available through telemedicine^46,47^ in BC, which can potentially help facilitate access, particularly in smaller communities with fewer primary care practitioners; this highlights the crucial role for local pharmacies in dispensing mifepristone, particularly when telemedicine is used. Our results support prior studies’ findings that pharmacists rate dispensing mifepristone in community practice as highly acceptable^48,49^ and, after dispensing mifepristone, most experienced few issues.^18,19,48,50,51^ Pharmacists in Canada are no longer required to complete additional training to dispense mifepristone; however, resources and checklists are available through the Canadian Abortion Provider Support website.^52,53^

### Strengths and Limitations

We used a pragmatic mystery caller design (ie, a study evaluating real-life routine practice conditions), which simulated prescription access experiences and enabled our team to answer the relevant patient experience question: “Can a pharmacy fill a mifepristone prescription in 3 calendar days, regardless of whether they have the medication currently in stock?”^24,25,26^ Our design comprehensively emulated the patient experience of accessing mifepristone through community pharmacies by requesting referrals from pharmacies with stated inability to dispense mifepristone within 3 days and by cross-referencing the referral to check referral validity. Furthermore, our province-wide, population-based sample and high response rate (>98%) enhanced the credibility of our findings, and we used robust geographic analytic methods to evaluate travel times between population areas and pharmacy locations.^34^ While Canada was one of the first regions to remove restrictions on mifepristone provision and dispensing, others have now adopted similar frameworks^54,55^; therefore, our setting can provide crucial generalizable evidence on pharmacy dispensing practices.

While our study had the aforementioned strengths, our methods are also subject to some limitations. Our population estimates and indicators of inequity used area-level census data, not individual-level data and may be relatively poor proxies for assessing barriers to care. We report the results of one telephone interaction only in the summer months. Factors like weather (eg, snow or forest fires), staffing, shipping delays, or stock shortages could impact dispensing times. Exploring all aspects of patient access (ie, finding a practitioner, getting to appointments, follow-up, and experiencing stigma) is crucial, but was not the focus of our study. Likewise, understanding pharmacists’ experiences dispensing mifepristone or reasons for not maintaining current stock and/or not dispensing would identify opportunities to further expand access,^56^ but were beyond the scope of this study.

While abortion is legal across Canada, health care delivery is managed at the provincial level and uptake of medication abortion likely differs province to province.^10,57^ Thus, our findings may reflect system-level programs specific to BC. However, policies that strengthen between-pharmacy referral networks, alternative funding schemes for expired medications, or continued professional development for pharmacists (including expanded uptake of the Canadian Pharmacists Association’s medication abortion toolkit^58^) could be deployed to strengthen mifepristone access in BC and elsewhere. Because Canada’s model for mifepristone provision may be of interest to other settings,^54,55^ our study provides evidence for how provision through community pharmacies may unfold in this context.

## Conclusions

In this cross-sectional study of pharmacists in a setting where abortion is available as a routine health service and mifepristone is regulated as a routine prescription, we found that pharmacists have played a key role in providing geographically distributed access to medication abortion. Policies and initiatives to support pharmacy referral networks, particularly in urban areas, could further improve patient experience and access. Our findings may guide medication abortion service planning in global settings with evolving mifepristone and medication abortion regulatory frameworks.

## Notes

Supplement 1. eMethods. Supplemental Methods and Telephone Survey script eFigure 1. Study exclusions flowchart eTable 1. Valid referrals among all non-dispensing pharmacies (n=498) eTable 2. Referral details among non-dispensing pharmacies eTable 3. Timeliness of mifepristone dispensation (today) among dispensing pharmacies eTable 4. At least one mifepristone-dispensing pharmacy and mean proportion of available pharmacies within specified travel times of each dissemination area and in subgroups by urban-rural status eTable 5. Reproductive aged female population in DAs with at least one pharmacy within a 60-minute drive time eFigure 2. Closest dispensing pharmacy across all of BC (15m walk, 15m drive, 60m drive, no access, no pharmacy) eTable 6. Population of reproductive aged females in dissemination areas impacted by low density of mifepristone-dispensing pharmacies within 15m walk, 15-, 30- and 60- minute drive times eFigure 3. Proportion of pharmacies in British Columbia, Canada with mifepristone access within a 30m drive and in downtown Vancouver and Victoria within 15m walk eFigure 4. Interaction of local availability and areas with highest deprivation quintiles

Supplement 2. Data Sharing Statement
