# Supplementary material for: Mifepristone Access Through Community Pharmacies When Regulated as a Routine Prescription Medication
Source: JAMA Netw Open. 2025 Nov 6;8(11):e2542096. doi: 10.1001/jamanetworkopen.2025.42096 (PMC12593126; doi:10.1001/jamanetworkopen.2025.42096)
Supplement: Supplement 1. — eMethods. Supplemental Methods and Telephone Survey script eFigure 1. Study exclusions flowchart eTable 1. Valid referrals among all non-dispensing pharmacies (n=498) eTable 2. Referral details among non-dispensing pharmacies eTable 3. Timeliness of mifepristone dispensation (today) among dispensing pharmacies eTable 4. At least one mifepristone-dispensing pharmacy and mean proportion of available pharmacies within specified travel times of each dissemination area and in subgroups by urban-rural status eTable 5. Reproductive aged female population in DAs with at least one pharmacy within a 60-minute drive time eFigure 2. Closest dispensing pharmacy across all of BC (15m walk, 15m drive, 60m drive, no access, no pharmacy) eTable 6. Population of reproductive aged females in dissemination areas impacted by low density of mifepristone-dispensing pharmacies within 15m walk, 15-, 30- and 60- minute drive times eFigure 3. Proportion of pharmacies in British Columbia, Canada with mifepristone access within a 30m drive and in downtown Vancouver and Victoria within 15m walk eFigure 4. Interaction of local availability and areas with highest deprivation quintiles [file jamanetwopen-e2542096-s001.pdf]

Supplemental Online Content

Nethery E, Xu C, Chan SYC, et al. Mifepristone access through community pharmacies when regulated as a routine prescription medication. JAMA Netw. Open. 2025; 8(11):e2542096. doi:10.1001/jamanetworkopen.2025.42096

eMethods. Supplemental Methods and Telephone Survey script

eFigure 1. Study exclusions flowchart

eTable 1. Valid referrals among all non-dispensing pharmacies (n=498)

eTable 2. Referral details among non-dispensing pharmacies

eTable 3. Timeliness of mifepristone dispensation (today) among dispensing pharmacies

eTable 4. At least one mifepristone-dispensing pharmacy and mean proportion of available pharmacies within specified travel times of each dissemination area and in subgroups by urban-rural status

eTable 5. Reproductive aged female population in DAs with at least one pharmacy within a 60-minute drive time

eFigure 2. Closest dispensing pharmacy across all of BC (15m walk, 15m drive, 60m drive, no access, no pharmacy)

eTable 6. Population of reproductive aged females in dissemination areas impacted by low density of mifepristone-dispensing pharmacies within 15m walk, 15-, 30- and 60-minute drive times

eFigure 3. Proportion of pharmacies in British Columbia, Canada with mifepristone access within a 30m drive and in downtown Vancouver and Victoria within 15m walk

eFigure 4. Interaction of local availability and areas with highest deprivation quintiles

This supplemental material has been provided by the authors to give readers additional information about their work.

© 2025 Nethery E et al. *JAMA Netw. Open*.

eMethods. Supplemental Methods

Mystery caller survey protocols

Pharmacy calls were made during any day of the week between 0700 and 2300. The survey was conducted with the first pharmacy staff member who picked up the phone or to whom the surveyor was transferred. If the surveyor was unable to reach the pharmacy or placed on hold for more than five minutes, the call was logged as an attempt. Surveyors called back if the call was interrupted by what appeared to be an accidental hang-up or disconnect. Surveyors did not leave voicemails. If the surveyors were unable to reach a pharmacy staff member after five attempts during regular business hours, the pharmacy was considered non-responsive.

The initial question asked was "I have a prescription for mifepristone, is that something you can fill?" If the pharmacy was able to fill the prescription, follow-up questions asked about how long it would be until the patient could pick up the prescription. If the pharmacy was unable or unclear whether the prescription could be dispensed within 3 days, the surveyor asked specifically "I need to take it within 3 days. Can you get it by then?". If the pharmacy was unable to dispense within 3 days or unable to dispense at all, the surveyor requested a referral to a pharmacy that could dispense within 3 days ("Do you know any other pharmacies in the area that carry it?").

Telephone Survey Script for Physical Storefronts

[Record Pharmacy ID, date and time of call, caller initials, and pharmacy operation type]

1: Hi, I have a prescription for Mifegymiso. Is that something you can fill?

- *a) No, we don't stock/have that -> Go to 2*
- *b) No, we don't stock that but we can order it for you -> Go to 4*
- *c) No -> Go to 3*
- *d) Yes -> Go to 4*
- *e) When do you need it by? -> Go to 5*
- *f) Yes, when do you need it by? -> Go to 5*
- *g) No, we don't get/dispense that -> Go to 7*
- *h) No, but I think X pharmacy does (a proactive referral) -> Go to 8*

2: Oh, ok. Is it something you can get for me?

- *a) Yes -> Go to 4*
- *b) No, we don't get/dispense that -> Go to 7*
- *c) No, but I think X pharmacy can (a proactive referral) -> Go to 8*

3: Oh, would you be able to order it in?

- *a) Yes -> Go to 4*
- *b) No -> Go to 7*
- *c) No, but I think X pharmacy can/does (a proactive referral) -> Go to 8*

4: Okay great. If I bring in the prescription today, when is the soonest I could pick it up?

Record when (e.g., 3pm, tomorrow, Tuesday)

If a proactive referral to a different pharmacy is given -> Go to 8

If no proactive referral is given -> Go to 6

5: I need to take it within 3 days. Can you get it by then?

- *a) Ok, we can get it within 2-3 days -> Go to 6*
- *b) We cannot get this medication until X date -> Go to 9 and record number of days until the date provided.*

© 2025 Nethery E et al. *JAMA Netw. Open*.

If a proactive referral to a different pharmacy is given -> Go to 8

If no proactive referral is given -> Go to 9

6: Ok, I need to think about it, but I will call you back if I'd like to get it. Thanks very much for your time.

[End call and complete response entry]

7: Oh, ok. Do you know any pharmacies in the area that do carry it?

a) Yes-> Go to 8
b) No -> Go to 9

8: Great, which pharmacy was that? Could you give me the name and phone number?

a) Record name/phone number/address -> Go to 9

9: Ok. Thank you very much for your time. [End call and complete response entry]

Possible Questions from Staff and Caller Responses:

Staff Question: Which medication? Could you please spell the name?

Response: Spell out Mifegymiso.

Staff Question: Do you know the DIN?

Response: What is the DIN?

Staff Response: The Drug Identification Number.

Response: No, but I can quickly Google it (pause for ~5 seconds). The DIN is 02444038.

Staff Question: Are you a patient of the pharmacy?

Response: No, I haven't been there before.

Staff Question: Are you a BC resident?

Response: Yes, I am.

Staff Question: Do you have MSP?

Response: Yes, I do.

Staff Question: Would you like me to prepare this for you / order this for you?

Response: I need to think about it, but I will call you back if I'd like you to order it for me. Thanks very much for your time.

Staff Question: Anything about what the medication is for or counseling about the medication:

Response: Oh, I don’t want to discuss that over the phone, but would discuss that with the pharmacist when I pick up.

Staff Question: What are you taking it for?

Response: For an abortion.

Or

Oh, I don’t want to discuss that over the phone, but will discuss that with the pharmacist when I pick up.

Staff Question: Is this time-sensitive? or Did the physician tell you it is urgent?
Response: Yes, I need to take this within 3 days from now.

Staff Question: Where do you live?

Response: Oh, I’m nearby.

If asked again, response: Yeah, I’ll have no problem getting there. I have a car and can drive there.

If asked for a more specific location, response: City/town of pharmacy address

Staff Question: Anything about cost to the caller or coverage (e.g., “it’s expensive”, “you will have to pay for it before we order it”).

Response: Oh, I thought it was covered by MSP. I searched it up online.

eFigure 1. Study exclusions flowchart

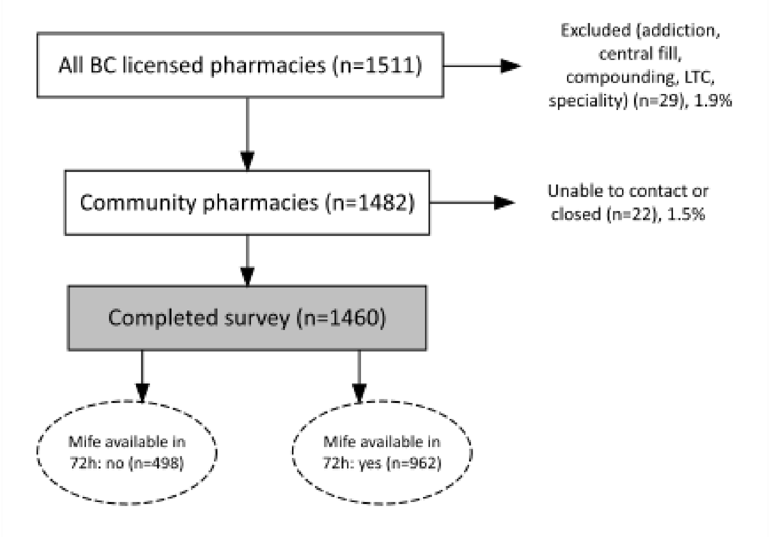

© 2025 Nethery E et al. *JAMA Netw. Open*.

eTable 1. Valid referrals among all non-dispensing pharmacies (n=498)

Characteristic	Proportion (%) with valid referrals (n/N) (95% CI) All non-dispensing pharmacies N = 498	Relative Risk (95% CI)	p-value
**Overall**	34% (169/498) 95%CI (30-38)		
**Pharmacy type**			
Corporate/chain	32% (45/141) 95%CI (24-40)	—	
Banner/franchise	35% (82/233) 95%CI (29-42)	1.10 (0.77-1.60)	0.60
Independent	34% (42/124) 95%CI (26-43)	1.06 (0.70-1.62)	0.78
**CHSA rural-urban type**			
Rural hub, rural or remote	39% (15/38) 95%CI (24-57)	—	
Medium or small urban	42% (44/106) 95%CI (32-51)	1.05 (0.60-1.95)	0.87
Metropolitan or large urban	31% (110/354) 95%CI (26-36)	0.79 (0.47-1.41)	0.38
**Region**			
Metro Vancouver	30% (92/302) 95%CI (25-36)	—	
Metro Victoria	30% (14/46) 95%CI (18-46)	1.00 (0.55-1.69)	>0.99
Medium cities	44% (37/85) 95%CI (33-55)	1.43 (0.96-2.07)	0.067
Other areas	38% (18/47) 95%CI (25-54)	1.26 (0.73-2.03)	0.37
Northern areas	44% (8/18) 95%CI (22-69)	1.46 (0.65-2.82)	0.31

Abbreviations: CI = Confidence Interval

© 2025 Nethery E et al. *JAMA Netw. Open*.

eTable 2. Referral details among non-dispensing pharmacies

Characteristic	All non-dispensing pharmacies N = 498[1]
Pharmacies without valid referral – Details of referral	329
no referral	209 (64)
referral to sexual health clinic	<5 (0.9)
specific referral to non-dispensing pharmacy	59 (18)
vague referral or call around	58 (18)
Valid referral (to a dispensing pharmacy)	169

[1]n (%)

© 2025 Nethery E et al. *JAMA Netw. Open*.

eTable 3. Timeliness of mifepristone dispensation among dispensing pharmacies

Characteristic	Can dispense today 356 / 962[1]	Relative Risk (95% CI)	p-value	Can dispense mifepristone today or tomorrow 808 / 962[2]	Relative Risk (95% CI)	p-value
**Overall**	37% (34-40)			84% (81-86)		
**Pharmacy type**						
Corporate/chain	34% (28-40)	—		74% (69-79)	—	
Banner/franchise	42% (37-46)	1.23 (0.97-1.59)	0.094	87% (84-90)	1.17 (0.99-1.39)	0.063
Independent	29% (23-36)	0.86 (0.61-1.19)	0.37	88% (82-92)	1.18 (0.96-1.45)	0.11
**CHSA rural-urban type**						
Rural hub, rural or remote	67% (58-75)	—		89% (82-94)	—	
Medium or small urban	50% (43-57)	0.74 (0.56-0.99)	0.040	84% (78-88)	0.94 (0.75-1.20)	0.64
Metropolitan or large urban	26% (23-30)	0.39 (0.30-0.51)	<0.001	83% (79-86)	0.93 (0.76-1.15)	0.49
**Region**						
Metro Vancouver	26% (22-30)	—		84% (80-87)	—	
Metro Victoria	18% (9.9-31)	0.71 (0.36-1.24)	0.27	73% (60-84)	0.87 (0.63-1.18)	0.40
Medium cities	48% (41-56)	1.86 (1.42-2.43)	<0.001	83% (76-88)	0.99 (0.82-1.19)	0.91
Other areas	65% (56-73)	2.51 (1.90-3.28)	<0.001	89% (81-93)	1.06 (0.85-1.30)	0.60
Northern areas	80% (64-90)	3.08 (2.07-4.46)	<0.001	88% (72-95)	1.04 (0.73-1.45)	0.81

[1]Proportion with mifepristone available today (%) among dispensing pharmacies (n/N) (95% CI)
[2]Proportion with mifepristone available today or tomorrow (%) among dispensing pharmacies (n/N) (95% CI)
Abbreviations: CI = Confidence Interval, RR = Relative Risk

© 2025 Nethery E et al. *JAMA Netw. Open*.

eTable 4. At least one mifepristone-dispensing pharmacy and mean proportion of available pharmacies within specified travel times of each dissemination area and in subgroups by urban-rural status

DAs with a pharmacy within:	At least 1 mifepristone dispensing pharmacy in this DA / total pharmacies in this travel distance n / N (%)[1]	Subgroup	Mean proportion of dispensing pharmacies in DA (95% CI)	RR this group compared to rural (95% CI)	P-value
15-min walk	5 269 / 5 688 (92.6%)	All	65.2 (64.5-65.9)		
		Rural hub, rural or remote	74.4 (71.4-77.4)	—	
		Medium or small urban	68.8 (67.0-70.6)	0.92 (0.91-0.94)	<0.001
		Metropolitan or large urban	63.0 (62.2-63.7)	0.85 (0.84-0.86)	<0.001
15-min drive	7 269 / 7 378 (98.5%)	All	66.1 (65.7-66.4)		
		Rural hub, rural or remote	73.7 (72.1-75.3)	—	<0.001
		Medium or small urban	67.9 (67.2-68.7)	0.92 (0.91-0.93)	<0.001
		Metropolitan or large urban	63.4 (63.2-63.6)	0.86 (0.85-0.87)	
30-min drive	7 450 / 7 514 (99.1%)	All	65.9 (65.6-66.2)		<0.001
		Rural hub, rural or remote	72.5 (71.2-73.8)	—	<0.001
		Medium or small urban	67.8 (67.2-68.4)	0.94 (0.93-0.94)	
		Metropolitan or large urban	63.2 (63.1-63.3)	0.87 (0.87-0.88)	
					<0.001
60-min drive	7 581 / 7 616 (99.5%)	All	66.1 (65.8-66.3)		<0.001
		Rural hub, rural or remote	70.8 (69.8-71.8)	—	
		Medium or small urban	68.8 (68.3-69.4)	0.97 (0.96-0.98)	
		Metropolitan or large urban	63.6 (63.5-63.7)	0.90 (0.89-0.91)	<0.001

1. 7848 total dissemination areas; 232 / 7 848 (3.0%) with no pharmacy in 60 minutes travel time.

© 2025 Nethery E et al. *JAMA Netw. Open*.

eTable 5. Reproductive aged female population in DAs with at least one pharmacy within a 60-minute drive time

Characteristic	At least 1 pharmacy within 60m drive, N = 1 110 218 (99.7%)	No pharmacy within 60m drive, N = 3 453 (0.31%)	Overall N = 1 113 671
All, n (%)			
yes	1 110 218 (100)	3 453 (0.3)	1 113 671
CHSA rural-urban type, n (%)			
Rural hub, rural or remote	122 841 (97)	3 385 (2.7)	126 226
Medium or small urban	186 094 (100)	68 (<0.1)	186 162
Metropolitan or large urban	801 283 (100)	0 (0)	801 283
Region, n (%)			
Metro Vancouver	709 959 (100)	29 (<0.1)	709 988
Metro Victoria	86 916 (100)	0 (0)	86 916
Medium cities	178 015 (100)	155 (<0.1)	178 170
Other areas	97 793 (98)	1 948 (2.0)	99 741
Northern areas	37 535 (97)	1 321 (3.4)	38 856
Census dissemination area type (recoded), n (%)			
City	844 865 (100)	0 (0)	844 865
District municipality	149 903 (100)	85 (<0.1)	149 988
Island municipality	770 (100)	0 (0)	770
Native govt	14 324 (85)	2 557 (15)	16 881
Regional district	74 574 (99)	726 (1.0)	75 300
Regional municipality	805 (98)	15 (1.8)	820
Town	15 681 (100)	0 (0)	15 681
Village	9 296 (99)	70 (0.7)	9 366

© 2025 Nethery E et al. *JAMA Netw. Open*.

eFigure 2. Closest dispensing pharmacy across all of BC (15m walk, 15m drive, 60m drive, no access, no pharmacy)

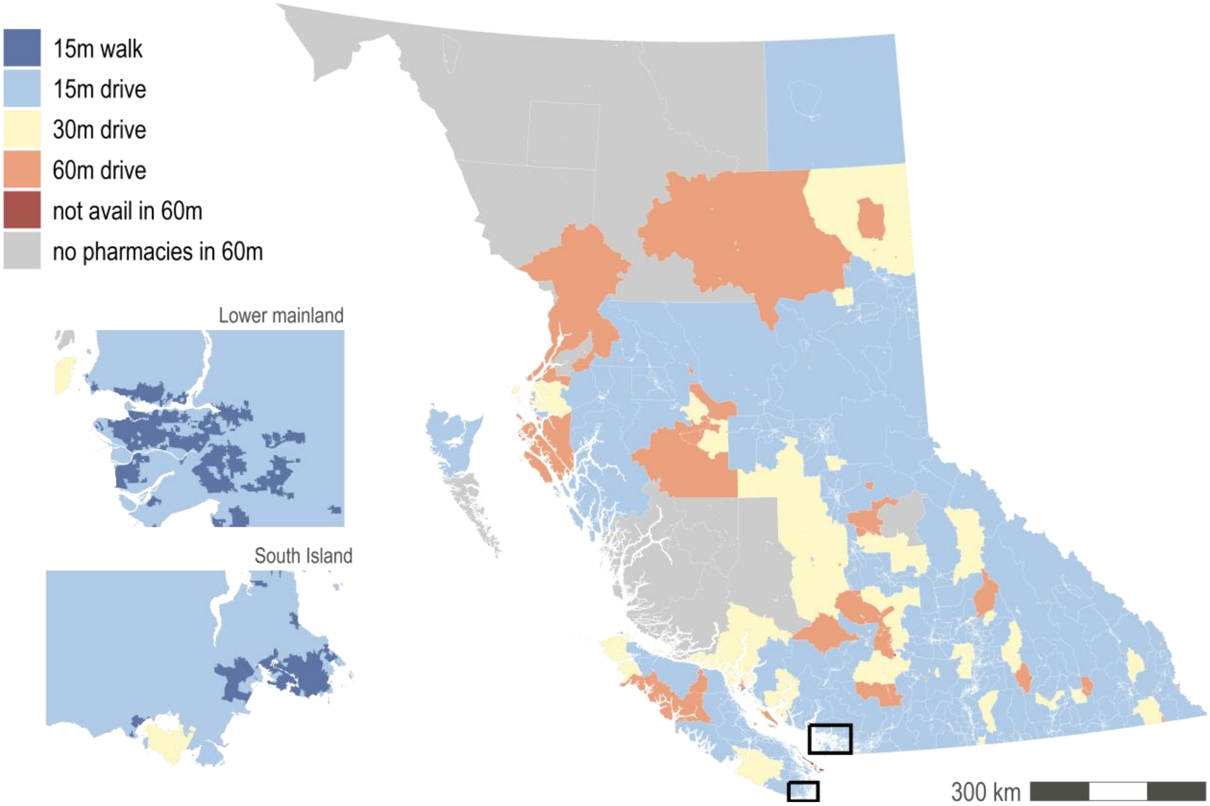

© 2025 Nethery E et al. *JAMA Netw. Open*.

eTable 6. Population of reproductive aged females in dissemination areas impacted by low density of mifepristone-dispensing pharmacies within 15m walk, 15-, 30- and 60- minute drive times

	Within 15min walk			Within 15min			Within 60min		
**Characteristic**	**Less than 50% of pharmacies in 15m walk 159 158 / 924 193 (17.2%)[1]**	**RR (95% CI)**	**p-value**	**Less than 50% of pharmacies in 15m drive 39 050 / 1 101 863 (3.54%)[1]**	**RR (95% CI)**	**p-value**	**Less than 50% of pharmacies in 60m drive 10 355/1 110 218 (0.93%)[1]**	**RR (95% CI)**	**p-value**
CHSA rural-urban type									
Rural hub, rural or remote	8768 (14)	—		9 486 (8.2)	—		5370 (4.4)	—	
Medium or small urban	25 011 (19)	1.29 (0.98 - 1.69)	0.069	11 934 (6.4)	0.78 (0.58 - 1.05)	0.10	4985 (2.7)	0.61 (0.40 - 0.94)	0.024
Metropolitan or large urban	125 379 (17)	1.19 (0.94 - 1.52)	0.15	17 630 (2.2)	0.27 (0.20 - 0.37)	<0.001	<5 (<0.1)	0.00 (0.00 - 0.00)	<0.001
Region									
Metro Vancouver	102 173 (16)	—		770 (0.1)	—		<5 (<0.1)	0.00 (0.00 - 0.00)	<0.001
Metro Victoria	24 496 (34)	2.12 (1.77 - 2.55)	<0.001	16 705 (19)	177 (67.4 - 468)	<0.001	10 (<0.1)	0.20 (0.01 - 3.13)	0.25
Medium cities	19 915 (16)	0.99 (0.81 - 1.21)	0.95	8 073 (4.6)	42.1 (15.3 - 116)	<0.001	105 (<0.1)	—	
Other areas	8328 (14)	0.91 (0.71 - 1.17)	0.46	9 359 (10)	92.6 (34.9 - 245)	<0.001	6362 (6.5)	110 (15.2 - 800)	<0.001
Northern areas	4246 (21)	1.30 (0.92 - 1.83)	0.14	4 143 (12)	107 (39.6 - 289)	<0.001	3878 (10)	175 (24.1 - 1,273)	<0.001
Economic dependency[2]									
least	77 170 (16)	—		13 035 (2.3)	—		2475 (0.4)	—	
most[3]	49 366 (20)	1.27 (1.10 - 1.48)	0.002	19 430 (6.1)	2.67 (1.95 - 3.65)	<0.001	6210 (1.9)	4.42 (2.67 - 7.30)	<0.001
Residential instability[5]									
least	34 985 (19)	—		10 775 (3.9)	—		2800 (1.0)	—	
most[3]	86 789 (17)	0.88 (0.74 - 1.04)	0.14	18 910 (3.5)	0.88 (0.63 - 1.24)	0.46	2820 (0.5)	0.51 (0.26 - 0.98)	0.042
Ethno-cultural composition[6]									
least	42 058 (18)	—		27 855 (8.1)	—		8295 (2.4)	—	
most[3]	84 577 (17)	0.93 (0.79 - 1.09)	0.35	445 (<0.1)	0.01 (0.00 - 0.03)	<0.001	230 (<0.1)	0.02 (0.00 - 0.07)	<0.001
Situational vulnerability[7]									
least	64 519 (17)	—		17 015 (3.6)	—		2970 (0.6)	—	
most[3]	58 907 (17)	0.99 (0.84 - 1.15)	0.87	15 980 (4.0)	1.11 (0.83 - 1.48)	0.47	6105 (1.5)	2.41 (1.36 - 4.27)	0.003

[1]n (%)
[2]Economic dependency relates to reliance on the workforce, or a dependence on sources of income other than employment income. (BC: proportion of the population who are aged 65 and older, the proportion of population participating in the labour force (aged 15 and older), the ratio of employment to population, the dependency ratio (population aged 0-14 and population aged 65 and older divided by population aged 15-64), and the proportion of children younger than age 6.)
[3]A value of 1 corresponds to dissemination areas that were the least deprived for that dimension, and a value of 5 corresponds to DAs that were the most deprived. Note that depending on an area's characteristics, it could be the most deprived for one dimension and the least deprived for another.
[4]Canadian index of multiple deprivation index data is not available for some dissemination areas with small population sizes due to limited census data and privacy restrictions; therefore we cannot include these populations in our analysis
[5]Residential instability speaks to the tendency of neighbourhood inhabitants to fluctuate over time, taking into consideration both housing and familial characteristics. (BC: proportion of dwellings that are apartment buildings, the proportion of persons living alone, the proportion of dwellings that are owned, persons per square kilometer, and the proportion of movers within the past 5 years)

© 2025 Nethery E et al. *JAMA Netw. Open*.

[6]Ethno-cultural composition refers to the community make-up of immigrant populations. (BC: proportion of the population self-identified as visible minority, the proportion of population that is foreign-born, the proportion of population with no knowledge of either official language (linguistic isolation), the average number of persons per room, and the proportion of population with no religious affiliation.)
[7]Situational vulnerability speaks to variations in socio-demographic conditions in the areas of housing and education, while taking into account other demographic characteristics. (BC: proportion of the population identified as Indigenous, the proportion of the population aged 25-64 without a high-school diploma, the proportion of homes needing major repairs, the proportion of single parent families, median income, the median dollar value of dwelling, and the proportion of population that is self-employed)
Abbreviations: CI = Confidence Interval, RR = Relative Risk

© 2025 Nethery E et al. *JAMA Netw. Open*.

eFigure 3. Proportion of pharmacies in British Columbia, Canada with mifepristone access within a 30m drive and in downtown Vancouver and Victoria within 15m walk

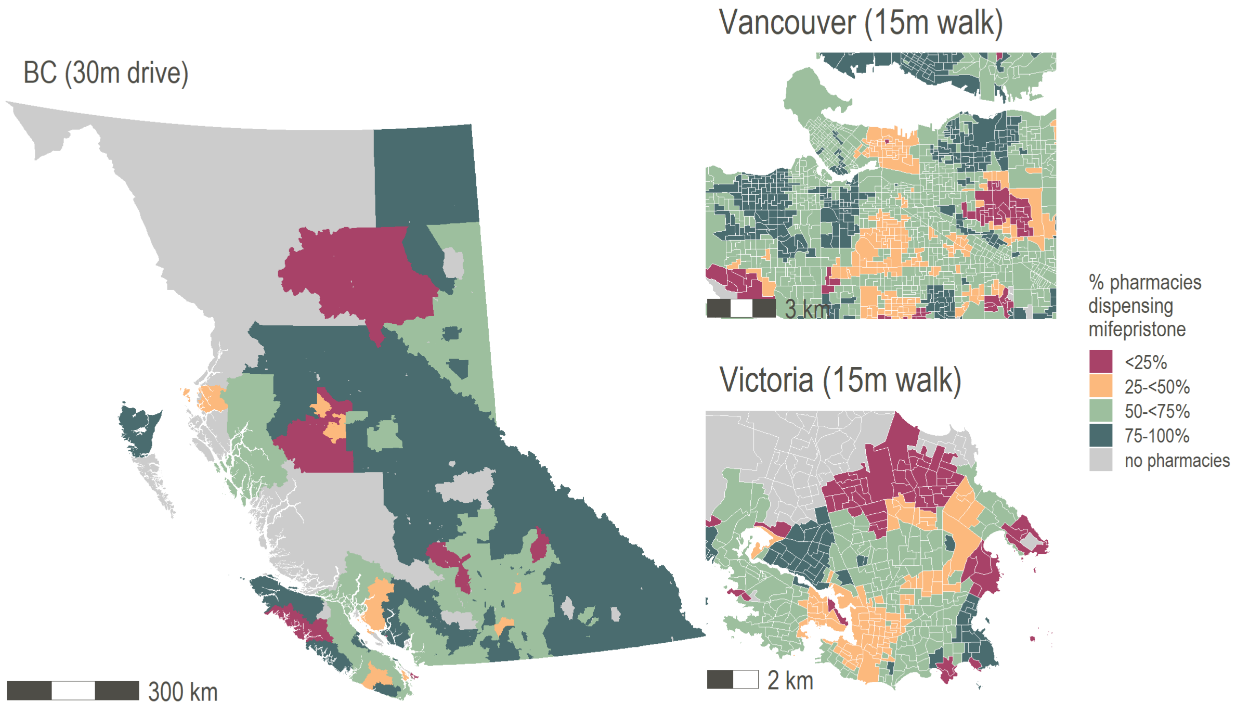

© 2025 Nethery E et al. *JAMA Netw. Open*.

eFigure 4. Interaction of local availability and areas with highest deprivation quintiles

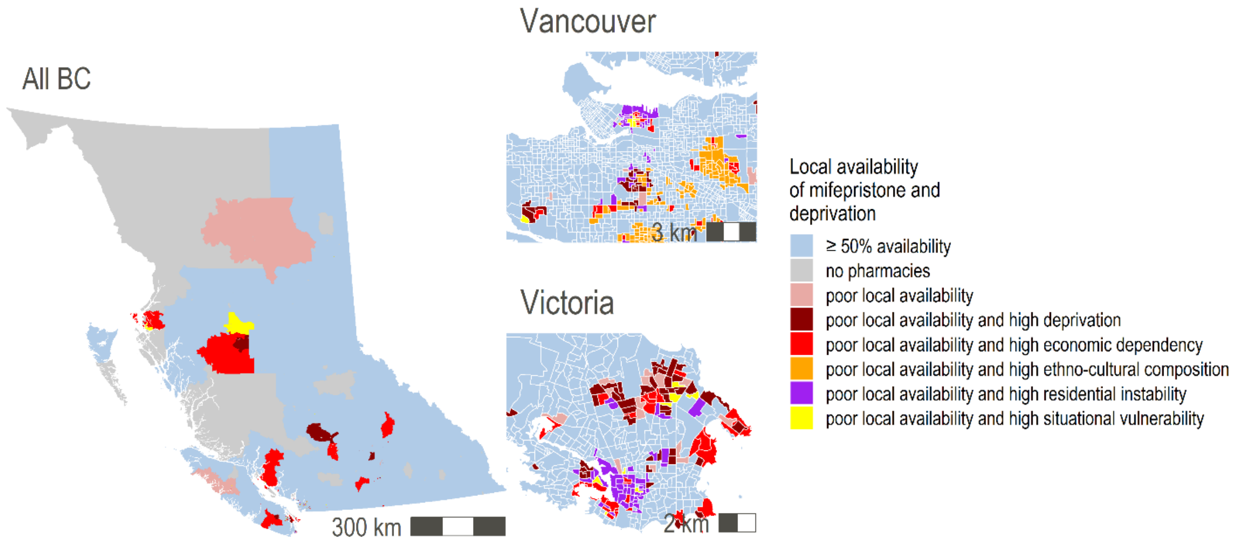

© 2025 Nethery E et al. *JAMA Netw. Open*.
