# Supplementary material for: Mifepristone Access Through Community Pharmacies When Regulated as a Routine Prescription Medication
Source: JAMA Netw Open. 2025 Nov 6;8(11):e2542096. doi: 10.1001/jamanetworkopen.2025.42096 (PMC12593126; doi:10.1001/jamanetworkopen.2025.42096)
Supplement: Supplement 2. — Data Sharing Statement [file jamanetwopen-e2542096-s002.pdf]

Data Sharing Statement

Nethery. Mifepristone Access Through Community Pharmacies When Regulated as a Routine Prescription Medication. *JAMA Netw Open*. Published November 06, 2025. doi:10.1001/jamanetworkopen.2025.42096

Data
Data available: No

Additional Information
Explanation for why data not available: To protect the identities of BC community pharmacies who were surveyed through the mystery caller survey, we cannot make this data available publicly.
